# Itraconazole as “bridge therapy” to anti-IgE in patients with severe asthma with fungal sensitization

**DOI:** 10.1186/2045-7022-3-S1-P28

**Published:** 2013-05-03

**Authors:** Stefano Pizzimenti, Enrico Heffler, Iuliana Badiu, Claudia Bussolino, Alberto Raie, Giovanni Rolla

**Affiliations:** 1 University of Torino - AO Mauriziano Umberto I, Allergy and Clinical Immunology, Italy

## Background

Sensitization to fungi has been reported to play an important role in a particular phenotype of severe asthma, the so called severe asthma with fungal sensitization (SAFS), characterized by high levels of total IgE, which may be an obstacle to anti-IgE therapy. A few studies showed the benefit of antifungal therapy in improving the quality of life of patients with SAFS associated to a decrease of total IgE serum concentration. We describe here the role of antifungal therapy as “bridge therapy”, which provided us the opportunity to start anti IgE therapy in one polysensitized patient with severe asthma, who had very high levels of total IgE, beyond the upper limits recommended for proper prescription of omalizumab.

## Method

A 59-year-old woman with uncontrolled severe asthma and frequent exacerbations, which required oral steroids courses, while on therapy with LABA / high dose inhaled fluticasone and montelukast, was evaluated for possible allergic broncho-pulmonary *aspergillosis* (ABPA).

## Results

The patient was sensitized to *Aspergillus umigatus*, house dust mites and grass pollen. She did not have *Aspergillus* precipitins and lung HRCT did not show bronchiectases or lung infiltrates, while she had very high levels of total IgE (1793 kUA/l), with specific grass, *D. Pter* and *A. fumigatus* IgE (respectively 15.3, 8.4 and 11.3 kUA/l). The patient received the diagnosis of SAFS and started itraconazole therapy (200 mg b.i.d.) as add-on therapy for 12 weeks. After treatment, a significant decrease of total IgE (1043 kUA/l) was found, associated to a mild improvement in asthma control (ACT from 16 to 20). At that time, omalizumab was started at recommended doses (300 mg every 2 weeks). During the six months after anti-IgE therapy the patient did not report any asthma exacerbation and ACT score (23) showed the good control of asthma.

## Conclusion

Antifungal therapy, as add-on treatment in patients with SAFS, may provide the opportunity to start anti-IgE therapy at usual recommended doses.

